# The impact of misclassifications and outliers on imputation methods

**DOI:** 10.1080/02664763.2024.2325969

**Published:** 2024-03-05

**Authors:** M. Templ, Markus Ulmer

**Affiliations:** a Institute for Competitiveness and Communication, School of Business, University of Applied Sciences and Art Northwestern Switzerland, Olten, Switzerland; b Institute of Data Analysis and Process Design, School of Engineering, Zurich University of Applied Sciences, Winterthur, Switzerland

**Keywords:** Missing values, imputation, simulation, outliers, misclassifications, robust methods, 62-08

## Abstract

Many imputation methods have been developed over the years and tested mostly under ideal settings. Surprisingly, there is no detailed research on how imputation methods perform when the idealized assumptions about the distribution of data and/or model assumptions are partly not fulfilled. This research looks into the susceptibility of imputation techniques, particularly in relation to outliers, misclassifications, and incorrect model specifications. This is crucial knowledge about how well the methods convince in everyday life because, in reality, conditions are usually not ideal, and model assumptions may not hold. The data may not fit the defined models well. Outliers distort the estimates, and misclassifications reduce the quality of most imputation methods. Several different evaluation measures are discussed, from comparing imputed values with true values or comparing certain statistics, from the performance of classifiers to the variance of estimated parameters. Some well-known imputation methods are compared based on real data and simulations. It turns out that robust conditional imputation methods outperform other methods for real data and simulation settings.

## 1. Introduction

Data often contains missing values, and the reasons for their appearance are manifold. When not properly treated, outliers might severely affect the imputation of missing values. We are left to ask: What methods are usually used for imputation, and do they work if the ideal conditions are not met?

The best choice of an imputation method should consider the population parameter to be estimated. In most cases, the data producer performs the imputation as one of the production steps (see, e.g. the Cross Industry Standard Process for Data Mining (CRISP) [8] or the General Statistics Business Production Model (GSBPM) [64] or other data mining models) without knowing the estimators of interest from other data users. Typically, data will be shared with many other people. Thus, generally, the producer of the data does not have knowledge of whether those who use the imputed data are attempting to calculate the average of a population or any other population parameter.

In doing so, one does not know whether the users of the imputed data are estimating the population mean or some other population parameter of interest. In this case, general purpose imputation methods are usually used, which we compare in the following. Note that further details can be found in [58].

*Imputation and multiple imputation*: Imputation replaces missing values in a data set with an estimated value by using the non-missing information in a data set. After this process, the data can be analyzed using standard techniques for complete data. To estimate the variance of the estimators properly, a multiple imputation procedure is usually used. Here, (single) imputation methods that contain a random component are applied more than once to a data set with missing values to create multiple copies of the imputed data sets. This approach makes it possible to properly estimate the variance of the estimated parameters using, for example, Rubin's rule to combine estimates [49].

*Kind of imputation methods*: Imputation methods range from model-free nearest neighbor and projection methods, whereby model-free means no formal statistical model is involved, to model-based and deep learning methods. Another distinctive feature of method groups is whether a joint distribution is modeled (joint modeling) or whether variables with missing values are modeled and imputed with multiple models (conditional modeling) sequentially. It has been demonstrated that joint and conditional modeling are equivalent when dealing with multivariate normal data. However, conditional modeling has distinct advantages when dealing with more intricate scenarios. For example, [66] mainly concentrates on conditional modeling for this purpose. Conditional modeling can be more robust than joint modeling, especially in the presence of model misspecifications. [52], for example, showed that conditional models used for multiple imputation can be (much) more robust than multiple imputation using joint modeling. Simulations from [33] have shown that this is especially true when categorical variables are included. Joint modeling approaches range from fitting multivariate distributions. These distributions can range from simple distributions to copulas (examine the applications of the ideas and implementation discussed in [13]) or deep learning approaches such as, for example, the implementation of generative adversarial neural networks to impute missing data in GAIN [69]. They can be successful for relatively simple structured data sets. However, the more variables and dependencies between the variables, the weaker a joint modeling approach becomes, and the stronger fully conditional modeling becomes. For a deeper discussion, see [58]. Obviously, the more complex the data and the more complex the relationships between variables, the more difficult it is to model a joint distribution well [2,58,66].

Generally, it is quite obvious that multiple and multivariate methods that make use of the correlations between variables are more appropriate than univariate imputation methods. Especially when the missing values are MAR (missing at random), univariate methods can be biased. MAR means that data are missing at random with respect to the value of the variable with missing values but not at random with respect to other variables. When missings occur randomly with respect to other variables and randomly with respect to occurrence in the variable of interest, we call the situation MCAR (missing completely at random).

*Evaluation of the quality of imputations*: To evaluate the quality of imputation methods, [10,59,61] proposed the use of visualization tools to compare the distribution of observed and imputed values. However, this is not always helpful. Observations with imputed values may not lie in the center of fully observed observations, nor might their distribution be similar, even though the imputation was excellent just because of a MAR situation. For example, when missing values in income occur only for old people and old people earn more than others, the imputed values should be in the upper tail of the observed income values. Unfortunately, situations can be more complex in a multivariate setting, and thus more formal measures of the quality of imputations come into focus. Many studies consider precision measures to compare imputed with known true values, for example, [28,62]. Others compare simple statistics like the mean, standard deviation, and correlation [40]. Bertsimas *et al.* [3], Poulos and Valle [43], Jäger *et al.* [29] and Woznica and Biecek [68] use complete data sets where all entries are known (“the ground truth”) and then generate patterns of missing data for different percentages of training data, impute the missing values in the training set, and then apply standard machine learning algorithms to obtain classification accuracy measures in a cross-validation setting.

*Simulation studies to evaluate the quality of imputations*: In addition, with more formal measures, properties such as unbiasedness and proper length of confidence intervals for imputed values can be investigated within a simulation [58]. Simulations to evaluate imputation methods for specific properties have been widely used in the literature. Some are carried out by repeatedly setting artificial missings in the complete part with the same mechanism as the missing values in the original data set [e.g. 30, 50, 62]. Others use a model to repeatedly simulate artificial data using a model with known parameters for data generation and missing value insertion. These parameters and their variance can then be estimated and compared with the true parameters. Such simulations are also frequently used to evaluate (multiple) imputation methods for unbiasedness in the variance estimation of parameters. It should be mentioned that almost all scientific evaluations and comparisons for judging variance estimates contain a very high percentage of missing values [e.g. 14, 26, 51, 65] in combination with simplified simulation settings. Generally, simulation studies are rare in this area with a missing rate of less than 50%, and usually only two- or three-dimensional data are generated without intricate structures, outliers, and misclassifications, and without data consisting of a mix of variables with different scales.

*Outliers and misclassifications*: The most important condition for single and multiple imputation of missing data is that the model used by the researcher to impute the missing values must be appropriate [58]. An investigation carried out by [57] revealed that existing imputation techniques have extreme difficulty in accurately filling in data when a significant interaction term is not included in the model. In practice, however, there might be outliers and/or – in the context of classifications – misclassifications in almost every data set.

Outliers can be categorized as representative, part of the population, or non-representative, caused by measurement errors [7]. Extreme values, another type of deviation, can also be observed. All these, including extreme values and outliers not caused by measurement errors, can significantly affect non-robust imputation methods, leading to poor imputation. This is particularly evident in statistical modeling, where leverage points can greatly influence the regression fit. Therefore, the impact of outliers on imputation methods is substantial, regardless of whether they are population outliers, measurement errors, or extreme values. In practice, it is often difficult to differentiate between these outlier types, but both types equally influence the imputation process.

Especially multivariate outliers are difficult to detect [27], require specialized expertize, and are a time consuming process [60]. It is not recommended to exclude outliers since it reduces the sample size [15] and thus introduces bias in variance estimation.

For multivariate missingness (missing values in multiple variables), conditional imputation methods are applied sequentially across variables. Although this automation simplifies method application, it does not ensure model assumption compliance or facilitate outlier and misclassification detection, which can be time consuming. A more effective approach is to employ robust imputation procedures that reduce the influence of outliers and/or misclassifications, preventing arbitrary impact on imputations.

*Contribution of this work*: We add the following points to the existing literature:

- A wide range of different methods is compared, from simple to non-linear methods, 10 methods in total.
- Evaluate common imputation methods under realistic assumptions about outliers, misclassifications, and model misspecifications.
- In the current literature, methods are mostly evaluated based on only one type of evaluation measure, and either they use real data sets or they evaluate methods in a simulation setting with model-based simulations. In this contribution, the evaluation of methods is based on five different types of evaluation measures, namely (1) visual comparisons using specific scatter plots including tolerance ellipses and results from the principal component analysis, (2) evaluation with precision measures by comparing imputed and true values and misclassifications, (3) comparison of correlation measures, (4) evaluation based on bias and variance of estimators including coverage rates, (5) evaluation in a classification context, and we use both real and simulated data sets.
- A more detailed description of the methods is provided than in other comparative studies.

*Outline*: In Section 2 selected imputation methods are presented. It is beyond the scope to consider all existing imputation methods, but it was the intention to select one of the most popular methods for each type of method. Section 3 introduces well-known evaluation methods to judge imputations and, thus, imputation methods. In Section 4, the data used in follow-up chapters is described, and the simulation setting is outlined. Section 5 shows all results compared to other studies in Section 6. Section 7 contains conclusions and recommendations.

## 2. Considered imputation methods

Before the actual methods are discussed, this paper will consider the particular features of imputation methods in terms of their use of *randomness*.

### 2.1. Randomness considered by imputation methods

Randomization to account for model uncertainty can involve fitting models on bootstrapped data or using Bayesian regression.

Imputation uncertainty and involved randomization, for example in a regression context, can include adding normal distribution values with zero mean and standard deviation equal to the model's residual standard error, sampling residuals with replacement, or bootstrap without replacement from the residuals, using closest donors in predictor space (PMM), or drawing residuals based on weighted probabilities (midastouch). For more details and a mathematical description, we refer to [58].

### 2.2. Simplified methods

The following two methods are only used as a reference point and should not be used in actual practice.

#### 2.2.1. Deletion of observations with missing values

This method refers to list-wise deletion, case deletion, complete case analysis, and available case analysis.

Deleting observations is often not recommended due to the considerable effort involved in data collection and the potential to introduce biases. Missing data, especially if randomly missing, can lead to inaccuracies in variance, standard error, and point estimates and also affect the power and degrees of freedom in statistical analyzes.

#### 2.2.2. Mean imputation

Mean imputation is when the missing value of a certain variable is replaced by the mean (e.g. arithmetic mean or median) of available cases. Without proof, we claim that this method is frequently used in practice and have therefore included it as a benchmark in our analysis. A multiple or multivariate imputation method should always outperform a simple mean imputation method, but as we will see later, this is not the case for all methods and also depends on the evaluation metrics chosen.

### 2.3. Imputation without a model

#### 2.3.1. Random hotdeck imputation

Hotdeck imputation replaces missing values in one observation (the receiver) with values from a similar (with respect to a distance measure) observation (the donor). Similar means that the receiver and the donor have similar values with respect to the observed part of the two observations. Random means that the procedure randomly imputes missing values from the available donors.

This method can be used for large data sets due to its computational efficiency and therefore remains a popular method.

The implementation in [61] is used due to the computationally efficient implementation [32].

#### 2.3.2. *k* nearest neighbor imputation

The *k* nearest-neighbor imputation method (*k*NN) is, without a doubt, one of the most popular imputation methods because of its simplicity.

It searches for the *k* nearest neighbors of the observation that contains the missing value. Note that there is no need to calculate all distances between all observations, but only for those that contain missings. These neighbors are chosen by the smallest Gower distance [24]. The mean value (e.g. arithmetic mean, median, mode) of the nearest neighbors *k* is used to impute the missing value.

The implementation in [32,61] is used since it is fast and provides a lot of features. This implementation supports weighting of variables (by the user or the random forest importance measure), randomness, conditions on donors, etc. In this work, sensible defaults are used: no weighting of variables, the median as a method for aggregating the *k* nearest neighbors in the case of numerical variables, the most frequent category in the case of categorical variables to be imputed, and *k*=5.

### 2.4. Linear model-based imputation

#### 2.4.1. Predictive mean matching (PMM) with mice

The standard PMM method uses Bayesian regression, i.e. it also takes model uncertainty into account and a stochastic matching distance. Regression methods (default is ordinary least squares regression) are not used to determine the actual imputed values, but are used to construct a metric for matching observations with missing values to similar, fully observed observations.

Instead of allowing all observations to be donors, standard predictive mean matching (PMM) restricts the number of potential donors for each value to be imputed. There are several options for how and how many donors are selected for the imputation of a missing value.

The standard PMM then does the following – in the univariate missingness situation – to impute missings in the variable y with *n* observations with fully observed predictor variables and corresponding model matrix X of dimension n×p and with (*obs*) and (*miss*) the index of observed and missing observations [see also 58, 65]. (*yobs*) and (*ymiss*) denote the observed and missing parts of variable y.

1. Apply a linear regression, $y_{\left(\mathrm{yobs}\right)}=X_{\left(\mathrm{yobs}\right)}\beta \;\beta +\epsilon_{\left(\mathrm{yobs}\right)}$, to estimate the unknown regression coefficients ββ.
2. Take a random draw from the posterior predictive distribution of $\hat{\beta \;\beta }$ using Bayesian regression to obtain new regression coefficients ${\hat{\beta \;\beta }}^{*}$. Typically, this would be a random draw from a multivariate normal distribution with mean $\hat{\beta \;\beta }$ and estimated covariance matrix of $\hat{\beta \;\beta }$.
3. Using ${\hat{\beta \;\beta }}^{*}$, generate predicted values for y, namely ${\hat{y}}^{*}$, for all *n* values, including those with missingness.
4. For $y_{\left(\mathrm{ymiss}\right)}$, that is, for each missing value, identify a set of *k* observations with observed $y_{\left(\mathrm{yobs}\right)}$ whose predicted values are close to the predicted value for the observations with missing data. Type 2: ${\hat{y}}^{*}=X_{\left(\mathrm{yobs}\right)}{\hat{\beta \;\beta }}^{*}$ matches ${\hat{y}}_{j}^{*}=X_{\left(\mathrm{ymiss}\right)}{\hat{\beta \;\beta }}^{*}$. Distances in the response space and not in the predictor space are considered.
5. Randomly select one of the nearby chosen candidates and assign its observed value as a substitute for the missing value.
6. For multiple imputation, repeat steps 2 to 5 m times to obtain the imputed data sets.

PMM, like many other methods, can be used when there are missing values in more than one variable using an expectation maximum algorithm. This is done by a sequential procedure in which imputation is performed variable by variable and by repetition until the imputed values stabilize.

In the following calculations, the implementation in R package mice [66] is used including the default settings with 5 multiple imputations and 5 donors are used. In the case of numeric response, predictive mean matching is used, while for categorical responses logistic and polytomous regression is used. For details on these two methods, we refer to [66].

#### 2.4.2. Midastouch

Midastouch represents a different approach to selecting candidate donors.

Siddique and Belin [53] proposed to sample one donor with a probability, where the probability depends on the distance between all donors and a recipient, thus all observed cases can serve as donors. Thus, the selection of the number of candidate donors is no longer needed. Gaffert *et al.* [22] enhanced this approach. Their proposed procedure is described in the following only for missings in one variable, but it can be generalized in an EM-based framework. Assuming only missings in y [see also 58, 65]:

1. Distances are calculated with $d_{\mathrm{ij}}=\left|\left(x_{i}-x_{j}\right)\cdot {\hat{\beta }}_{-i}^{*}\right|$, with $x_{i}$ the *i*th donor observation (where y has observed values), and $x_{j}$ the *j*th receipient (where y has a missing values). This corresponds to type 2 matching. ${\hat{\beta }}_{-i}^{*}$ is derived from bootstrapped $X_{\left(\mathrm{yobs}\right)}$ data.
2. Compute the probabilities of a recipient *j* receiving donor *i* from the full donor pool based on weighted distances $P(i\overset{\mathrm{imputes}}{\to }j)=f\left(\omega \;\omega ,{\hat{y\;y}}_{\mathrm{yobs}}^{*},{\hat{y\;y}}_{j\in \mathrm{ymiss}},\kappa \right)=\frac{\omega_{i}\cdot d_{i,j}^{-\kappa }}{\sum_{i=1}^{n_{\mathrm{obs}}}\left(\omega_{i}\cdot d_{i,j}^{-\kappa }\right)}$ with ${\hat{y\;y}}_{\mathrm{yobs}}^{*}$ derived from bootstrapped data, more precisely from an approximate Bayesian Bootstrap [for details see 22]. *κ* expresses the importance of a distance and the weights ωω are bootstrap frequencies of the donors; see [22].
3. Estimate *κ* with $\kappa \left(R_{\mathrm{obs}}^{2}\right)={\left(\frac{50\cdot R_{\mathrm{obs}}^{2}}{1+\delta -R_{\mathrm{obs}}^{2}}\right)}^{3/8}$ where $R_{\mathrm{obs}}^{2}$ is the coefficient of determination of the model $y_{\left(\mathrm{yobs}\right)}=X_{\left(\mathrm{yobs}\right)}\beta \;\beta +\epsilon \;\epsilon_{\left(\mathrm{yobs}\right)}$. The larger the $R^{2}$ the more important the distances in (2). When $R^{2}$ is about 0.9 then *κ* is about 10.
4. Take the observed value in y for the randomly selected donor as the imputation of a missing value.
5. Repeat steps 1 to 4 to impute all missing values in $y_{\left(\mathrm{ymiss}\right)}$.
6. Repeat the whole procedure *m* times to produce *m* multiple imputed data sets.

Furthermore, the too low variability of the imputed data sets *m* is corrected by the Parzen method [41]. Again, in the implementation in the R package mice midastouch is used for numerical responses, but logistic and polytomous regression are used for categorical responses.

#### 2.4.3. Robust imputation with IRMI

A common assumption for multivariate imputation methods is that the data come from a generating distribution (e.g. multivariate normal) or that the data can be transformed to it. This is not the case if the data contains outliers. In this case, standard methods can lead to imputed values that are far away from the distribution of regular, observed observations, and rather robust statistical procedures should be used.

IRMI (iterative robust model-based imputation) [62] allows EM-based imputation with robust methods for mixed-scaled variables. For details of robust methods for count, binary, and categorical responses, see [6]. The algorithm implemented in the R package VIM in the function irmi works as follows [see also 58, 62]:

Step 1: Initialization of the missing values by, e.g. *k*-nearest neighbor imputation.
Step 2: Sort the variables according to the original number of missing values. We now assume that the variables are already sorted, that is, $M(x\;x_{1})\geq M(x\;x_{2})\geq \cdots \geq M(x\;x_{p})$, where $M(x\;x_{j})$ denotes the number of missing cells in variable $x\;x_{j}$. Set I={1,…,p}.
Step 3: Set *l*=1.
Step 4: Denote $m_{l}\subset \{1,\ldots ,n\}$ the indices of the observations that were originally missing in variable $x\;x_{l}$, and $o_{l}=\{1,\ldots ,n\}\setminus m_{l}$ the indices corresponding to the observed cells of $x\;x_{l}$. Let $X\;X_{I\setminus \{l\}}^{\left(o_{l}\right)}$ and $X\;X_{I\setminus \{l\}}^{\left(m_{l}\right)}$ denote the matrices with the variables corresponding to the observed and missing cells of $x\;x_{l}$, respectively. Additionally, the first column of $X\;X_{I\setminus \{l\}}^{\left(o_{l}\right)}$ and $X\;X_{I\setminus \{l\}}^{\left(m_{l}\right)}$ consists of ones that take care of an intercept term in the regression problem
(1) $$\begin{array}{r} x\;x_{l}^{\left(o_{l}\right)}=X\;X_{I\setminus \{l\}}^{\left(o_{l}\right)}\beta \;\beta +\varepsilon \;\varepsilon \end{array}$$
  with unknown regression coefficients ββ and an error term εε. Note that $X\;X_{I\setminus \{l\}}$ contains only the remaining variables by default. The user can also define a separate model for each of the *l*th variables selected in the inner loop, then $X\;X_{I\setminus \{l\}}$ denotes the model matrix. In this way, the user can define the transformation of variables, etc.
  The distribution of the response $x\;x_{l}^{\left(o_{l}\right)}$ is considered in each regression fit. If the response is
  - *continuous*, the link is μμ and a robust regression method (see below) is applied;
  - *categorical*, generalized linear regression is applied (optionally, a robust method may be selected);
  - *binary*, the link is $\log(\frac{\mu_{i}}{1-\mu_{i}})$, for i=1,…,n, i.e. logistic linear regression is applied (optionally, a robust method can be selected);
  - *semi-continuous*, a two-stage approach is used, where in the first stage logistic regression is applied (default: classical logistic regression) in order to decide if a constant (usually zero) is imputed or not. In the latter case, the imputation is done by robust regression based on the continuous (non-constant) part of the response.
  - *count*, robust generalized linear regression of family Poisson is used with link $\log(\mu_{i})$, for i=1,…,n.
  Optionally, it is possible to use a stepwise model selection by AIC (parameter step in function irmi) to include only the *k* most important variables, k⊂I∖{l}, in the regression problem above. Otherwise, k=I∖{l}.
Estimate the regression coefficients ββ with the corresponding model in Step 4, and use the estimated regression coefficients $\hat{\beta \;\beta }$ to replace missing parts $x\;x_{l}^{\left(m_{l}\right)}$ by
(2) $$\begin{array}{r} {\hat{x\;x}}_{l}^{\left(m_{l}\right)}=X\;X_{k}^{\left(m_{l}\right)}\hat{\beta \;\beta }. \end{array}$$
Carry out Steps 4–5 in turn for each l=2,…,p.
Repeat Steps 3–6 until the imputed values stabilize, i.e. until
$$\begin{array}{r} \sum_{i}({\hat{x\;x}}_{l,i}^{\left(m_{l}\right)}-{\tilde{x\;x}}_{l,i}^{\left(m_{l}\right)}{)}^{2}<\delta ,\;\text{for all}\;i\in m_{l}\text{ and }l\in I, \end{array}$$
  for a small constant *δ*, where ${\hat{x\;x}}_{l,i}^{\left(m_{l}\right)}$ is the *i*th imputed value of the current iteration, and ${\tilde{x\;x}}_{l,i}^{\left(m_{l}\right)}$ is the *i*th imputed value from the previous iteration.
Repeat once Step 3–Step 5, but in step 5 replace the missing parts with ${\hat{x\;x}}_{l}^{\left(m_{l}\right)}=X\;X_{k}^{\left(m_{l}\right)}\hat{\beta \;\beta }+{\hat{\epsilon }}^{*}\cdot \sqrt{1+\frac{1}{n}\#m^{l}}$. The error term ${\hat{\epsilon }}^{*}$ has mean 0 and variance corresponding to the robust variance of the regression residuals $\hat{\epsilon }$. The constant $\sqrt{1+\frac{1}{n}\#m^{l}}$ takes into account the number of missing values ( $\#m^{l}$) in the response.

Regarding randomness, this approach is type 1 and takes only the imputation uncertainty into account, but it can also be easily extended to type 2 [57]. IRMI is implemented in R package VIM [32]. A variant and experimental version of IRMI that allows complex models for each variable to be specified, PMM and midastouch for the imputation uncertainty, and a robust bootstrap for the model uncertainty are available in [57].

### 2.5. Non-linear methods

In case the transformation of variables or the inclusion of interactions does not linearize the dependency between the response and predictions using model-based imputation, non-linear methods are preferable.

#### 2.5.1. Imputation using random forests

First, the missing values are initialized for random forest imputation, and with the completed data set, a random forest is fitted.

Set I={1,…,p} with *p* the number of variables in the matrix X and let $p_{\left(\mathrm{cont}\right)}$ and $p_{\left(\mathrm{cat}\right)}$ the indices of the continuous variables ( $p_{\left(\mathrm{cont}\right)}=I_{\left(\mathrm{cont}\right)}\subseteq I$ and $p_{\left(\mathrm{cat}\right)}=I_{\left(\mathrm{cat}\right)}\subseteq I$) and categorical variables, respectively. The algorithm works as follows [see also 39, 55, 58]:

Step 1: Initialize the missing values using a simple imputation technique, i.e. mean imputation.
Step 2: Set *l*=1.
Step 3: Denote again $m_{l}\subset \{1,\ldots ,n\}$ the indices of the observations that were originally missing in variable $x_{l}$ and $o_{l}=\{1,\ldots ,n\}\setminus m_{l}$ the indices corresponding to the observed cells of $x_{l}$. $|m_{l}|$ defines the number of missing values in variable *l*. Let $X_{I\setminus \{l\}}^{o_{l}}$ and $X_{I\setminus \{l\}}^{m_{l}}$ denote the matrices with the variables corresponding to the observed and missing cells of $x_{l}$, respectively. For the fitting and prediction step, sort the variables in $X_{I\setminus \{l\}}$ according to the original amount of missing values. Afterwards, sort the variables back to the previous order.
Step 4: Fit the random forest: $x_{l}^{o_{l}}∼X_{I\setminus \{l\}}^{o_{l}}$
Step 5: Predict ${\hat{x}}_{l}^{m_{l}}$ using $X_{k}^{m_{l}}$ and replace/update the missing parts $x_{l}^{m_{l}}$
Step 6: Carry out Steps 4–5 in turn for each l=2,…,p.
Step 7: Repeat steps 3–6 until the imputed values stabilize, i.e.
  - for each continuous variable this holds
$$\begin{array}{r} \frac{\sum_{i}({\hat{x}}_{l,i}^{m_{l}}-{\tilde{x}}_{l,i}^{m_{l}}{)}^{2}}{\sum_{i}\left({\hat{x}}_{l,i}^{m_{l}}\right)}<\delta ,\;\text{for all}\;i\in m_{l}\text{ and }l\in I_{\left(\mathrm{cont}\right)}, \end{array}$$
  - for each categorical variable this holds
$$\begin{array}{r} \frac{\sum_{i}I({\hat{x}}_{l,i}^{m_{l}}\neq {\tilde{x}}_{l,i}^{m_{l}})}{|m_{l}|}<\delta ,\;\text{for all}\;i\in m_{l}\text{ and }l\in I_{\left(\mathrm{cat}\right)}, \end{array}$$
  for a small constant *δ*, where ${\hat{x}}_{l,i}^{m_{l}}$ is the *i*th imputed value of the current iteration, and ${\tilde{x}}_{l,i}^{m_{l}}$ is the *i*th imputed value from the previous iteration. *I* defines the indicator function, which has values of 1 when the condition is true, otherwise 0.

Step 5 can be modified to allow for predictive mean matching.

Random forest imputation method is available in R package missForest [55] and R package ranger [39]. The latter one is the preferred implementation, as it is more efficient in terms of computational speed, less error-prone, and contains more validity checks.

#### 2.5.2. Imputation using XGBoost

Ensemble methods like Xtreme Gradient Boosting (XGBoost) [9] are popular in data science for their robust performance, with XGBoost being a notable distributed gradient boosting algorithm. It improves upon previous methods by subsampling with replacement and random feature selection, and like random forests, is resistant to overfitting. Unlike random forests, which build independent trees, XGBoost constructs successive decision trees to correct preceding errors. It is applicable for both classification and regression trees. More details are in [12], and the algorithm can sequentially impute missing values across variables, with multiple imputations possible through repeated procedure calls. When multiple variables have missing values, they are sorted by ascending order of missing values.

Step 1: Initialize the missing values using a simple procedure.
Step 2: Generate a bootstrap sample and fit a XGBoost model. This is done sequentially for all variables.
Step 3: Predict the missing values for each variable using the model fitted on the bootstrap sample and the observed values of the original data.
Step 4: Match the observations with missing values in the variable to be imputed, that is, apply a predictive mean matching procedure for imputation.

#### 2.5.3. Imputation using deepImp

Several proposals suggest the use of deep learning methods, particularly generative adversarial networks, for joint modeling [34,37,69]. However, these approaches have limitations, including the restriction to continuous data and the non-production readiness of existing implementations in Python [69] and R [34]. The suggested conditional modeling approach iteratively employs deep artificial neural networks, where the output layer structure, activation function, loss, and evaluation function depend on the assumed variable distribution. This algorithm operates in a chain, being applied repeatedly to each variable until convergence, similar to an EM-based method.

Let $X=(x_{1},x_{2},\ldots ,x_{p})$ be a n×D-dimensional data matrix. $x_{k}^{\left(\mathrm{obs}\right)}$ denotes the observed values of the *k*th variable, while $x_{k}^{\left(\mathrm{miss}\right)}$ represents the missing values.

The method works as follows [56].

Step 1: Initialize the missing values using another imputation technique, such as *k*NN imputation. Let $X^{\left(\mathrm{imp}\right)}$ the imputed data matrix.
Step 2: Set r←0, the index for iterations and *maxiter* the maximum number of iterations.
Step 3: Calculate k, the vector of indices with columns in X that contain missing values, and *m*, the number of missing values in X.
Step 4: *r*=*r*+1
Step 5: Store previously imputed data matrix $X^{\left(\mathrm{imp},\mathrm{old}\right)}$
Step 6: for j in 1:p do
  Fit the deep neural net: $x_{s}∼(x_{1},x_{2},\ldots ,x_{D})\setminus x_{s}$ and predict $x_{s}^{\left(\mathrm{miss}\right)}$ $X^{\left(\mathrm{imp},\mathrm{new}\right)}\leftarrow$ update imputed data matrix with $x_{s}^{\left(\mathrm{miss}\right)}$
Step 7: Update $\Delta =\frac{1}{m}|X^{\left(\mathrm{imp},\mathrm{new}\right)}-X^{\left(\mathrm{imp},\mathrm{old}\right)}|$
Step 8: Until Δ>ϵ and *r*<*maxiter* go to Step 4.
Step 9: Update $X^{\left(\mathrm{imp}\right)}=X^{\left(\mathrm{imp},\mathrm{new}\right)}$

Hyperparameters of the artificial neural networks fitted should be chosen with care. To prevent overfitting or underfitting, it is recommended to at least evaluate whether overfitting or underfitting occurs with a selected hyperparameter setting by comparing the training error and the validation error in a cross-validation setting. These errors should be approximately equal.

Usually, 2–3 iterations are sufficient (*maxiter*). Specifying all the details on the stopping criteria would break the page limits of this article, but we refer to [56].

In the deep neural network, the dataset is processed through multiple epochs, with 200–300 epochs usually providing satisfactory results. To prevent overfitting, we employ a stopping criterion based on the ‘patient value’, halting when weights remain stable for a set number of epochs (35 in our case), and a 10% dropout rate in the first half of the layers to ensure diverse re-imputation results. Layer-wise, too many can lead to overfitting and too few to underfitting. For moderate to large datasets, starting with 1000 neurons and decreasing by 100 per layer up to the 10th layer is effective, while for smaller datasets, configurations of 300, 128, 64, and 32 neurons are used across four layers.

For the optimizer, our default choice is Adam [31], a first-order stochastic gradient-based optimization based on adaptive estimates of lower-order moments.

The choice of loss function depends primarily on the type of scale of the response variable (the variable to be imputed). For nominal variables to be imputed, a cross-entropy measure is usually used, while for continuous variables, a mean square error is used. For the evaluation metrics that assess the performance of the model, the mean absolute error is chosen. To allow for non-linear combination of neurons, we choose by default the activation *reLU*, see [67]. For the last layer (which actually does the imputation), a linear activation must be used in case of continuous response to impute and *softmax* for a categorical response.

## 3. Evaluation methods and measures

### 3.1. Evaluating the imputed values

#### 3.1.1. Evaluation through scatterplots

**Figure 1.**
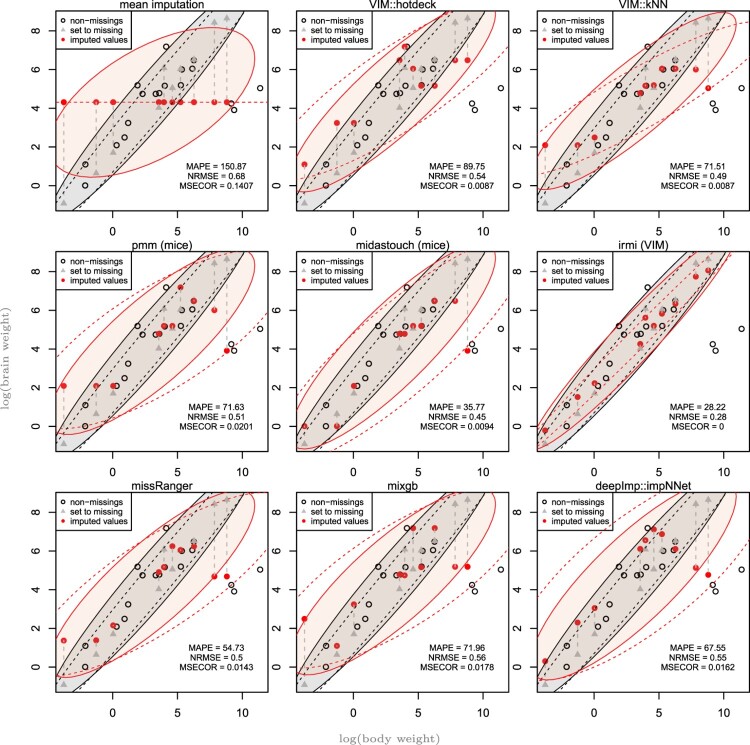
Results for the Animals data set. True values set to missing (grey triangles), imputed values (red points) and their connecting lines to the true values (dashed gray lines), robust tolerance ellipses of the complete data (ellipses in solid black lines) and after imputation (ellipses in black dashed lines) and the subset of values to be imputed (ellipses in solid red for true values and in dashed red lines for imputed values).

This is for illustrations of bivariate continuous distributions. From the complete data, some values are marked as missing, and the values are afterwards imputed. With this setting, we know the true values from the complete data and the results of imputing these artificial sets of missing values. We show one realization, and repeating the imputations would result in slightly different results/graphics. However, a general picture can be drawn of how the methods may perform. What exactly do you see in this illustration? In Figure 1, you see, on the one hand, the complete data (black circles and gray triangles), those data values that were set to missing (regarding their *y* values, gray triangles) and their imputation (red-filled circles). In addition, robust covariances are shown in the form of tolerance ellipses [46]. More precisely:

- Robust 95% tolerance ellipses are shown for both imputed and complete original data (solid lines and shaded areas, black for original data, red for imputed data). Essentially, the multivariate structure of the original data is compared with the imputed data.
- It also compares the robust tolerance ellipses of the missing part of the original complete data and the same subset of the imputed data. So here we compare only the missing part and see if the observed values line with the imputed values, so we see a more accurate slice of the data with missings and imputations.

#### 3.1.2. Evaluation through biplots

A biplot [21] provides a powerful visual tool for visualizing the results of principal component analysis. It can be used to (1) visually analyze whether the imputations went well and (2) compare the imputation methods for missing values.

In a biplot for principal components, only *k* columns of scores U and loadings B are used, in most cases 2. The scores U contain the information about the objects and the loadings B contain the information about the variables.

Generally, in a biplot, this holds:

- The orthogonal projections of the observations onto the variable(s) (arrows) approximate the original (centered and possibly standardized) data values.
- The cosine of the angle between the variable(s) (arrows) approximates the correlation between the variables.
- The Euclidean distances between the observations approximate the Mahalanobis distances (= multivariate distances) of these observations. If the data were scaled before applying PCA, the Mahalanobis distances of the scaled observations are approximated.

To analyze the imputed values and thus assess (or compare) imputation methods for a given data set, the PCA scores of the imputed values can be highlighted in a biplot to see their appearance compared to the PCA scores of the fully observed observations. Of interest is using the fully observed data and randomly inserting some missing values. Then these artificially created missing values are imputed. By comparing two biplots, one from the complete data and one from the same but imputed data after inserting missing values, we can observe how well an imputation method works.

Additionally, the first two loading vectors, say $B_{\left[p\times 2\right]}$, which are obtained from a principal component analysis of the fully observed complete data, are compared to the one obtained from insertion and imputation of missing values, say $B_{\left[p\times 2\right]}^{\left(\mathrm{imp}\right)}$ by (in percentage)

(3) $$\begin{array}{r} \mathrm{rel}.\mathrm{diff}.\left(B_{\left[p\times 2\right]},B_{\left[p\times 2\right]}^{\left(\mathrm{imp}\right)}\right)=\frac{1}{2p}\sum_{j=1}^{2}\sum_{i=1}^{p}\left|\frac{b_{\mathrm{ij}}-b_{\mathrm{ij}}^{\left(\mathrm{imp}\right)}}{b_{\mathrm{ij}}}\right| \end{array}$$

### 3.2. Evaluation with precision measures

Either in a simulation setting or for the complete part of real data sets, known values are set to missing and imputed afterwards. Generally, for examples with precision measures, we use 10% MCAR in each variable of a data set and repeat the procedure of setting missing values and imputing them 1000 times.

#### 3.2.1. Mean absolute percentage error (MAPE)

The MAPE is useful in simulations or when extra missing values are added to a dataset, assessing the accuracy of imputations against true values. While this measure provides valuable precision insights, it doesn't fully capture imputation's true essence, as it doesn't reflect the uncertainty of imputed values, which is later addressed by the coverage rate.

Let $x_{j}^{\left(\mathrm{imp}\right)}$ be the imputed column of X, and $x_{j}^{\left(\mathrm{orig}\right)}$ the same normalized column, containing the values that originally should have been observed.

The MAPE for continuous variables of a data set is then estimated by

(4) $$\begin{array}{r} \mathrm{MAPE}(X^{\left(\mathrm{imp}\right)},X^{\left(\mathrm{orig}\right)})=100\cdot \frac{1}{\sum_{i=1}^{n}\sum_{j\subseteq I_{\left(\mathrm{cont}\right)}}m_{\mathrm{ij}}}\sum_{i=1}^{n}\sum_{j\subseteq I_{\left(\mathrm{cont}\right)}}\frac{|x_{\mathrm{ij}}^{\left(\mathrm{imp}\right)}-x_{\mathrm{ij}}^{\left(\mathrm{orig}\right)}|}{x_{\mathrm{ij}}^{\left(\mathrm{orig}\right)}} \end{array}$$

with $m_{\mathrm{ij}}$ the cells of the indicator matrix M containing the information if an element is missing or not (in case of a missing value, it equals 1, otherwise 0). The notation $j\subseteq I_{\left(\mathrm{cont}\right)}$ should emphasize that only continuous measurements variables are considered in this equation, although the MAPE can also be computed for mixed variables in case Gower distances are used.

#### 3.2.2. Normalized mean root squared error (NRMSE)

For continuous variables in a data set, this is defined as [see also, e.g. 55]

(5) $$\begin{array}{r} \mathrm{NRMSE}(X^{\left(\mathrm{imp}\right)},X^{\left(\mathrm{obs}\right)})=\frac{1}{\sum_{i=1}^{n}\sum_{j\subseteq I_{\left(\mathrm{cont}\right)}}m_{\mathrm{ij}}}\sum_{i=1}^{n}\sum_{j\subseteq I_{\left(\mathrm{cont}\right)}}\frac{(x_{\mathrm{ij}}^{\left(\mathrm{imp}\right)}-x_{\mathrm{ij}}^{\left(\mathrm{orig}\right)}{)}^{2}}{{s^{2}}_{j}^{\left(\mathrm{orig}\right)}} \end{array}$$

with

$$\begin{array}{r} s_{j}^{2\;(\mathrm{orig})}=\frac{1}{\sum_{i=1}^{n}m_{\mathrm{ij}}-1}\sum_{i=1}^{n}(x_{\mathrm{ij}}^{\left(\mathrm{orig}\right)}-{\bar{x}}_{j}^{\left(\mathrm{orig}\right)}{)}^{2}. \end{array}$$

A good performance is given at a value close to 0, and a poor performance is at a value around 1.

#### 3.2.3. Differences in correlations (MSECOR)

The influence of the imputation on the multivariate data structure is here expressed by the difference in correlation structure.

Let $R=[r_{\mathrm{ii}}]$ be the sample correlation matrix of the original observations. Further, $\tilde{R}=[{\tilde{r}}_{\mathrm{ij}}]$ denotes the sample correlation matrix computed where all missing cells have been imputed. The difference in the correlation structure is based on the Euclidean distance between the two correlation estimates, as

(6) $$\begin{array}{r} \mathrm{MSECOR}=\sqrt{\frac{1}{(D-1{)}^{2}}\sum_{i=1}^{D-1}\sum_{j=1}^{D-1}{\left(r_{\mathrm{ij}}-{\tilde{r}}_{\mathrm{ij}}\right)}^{2}}=\frac{1}{D-1}\|R-\tilde{R}\| \end{array}$$

For simulations with certain observations selected as outliers, the measure should only be computed for the non-outliers.

#### 3.2.4. False classifications (FC)

The equivalent error measurement for categorical and binary variables is the percentage of the imputed values that are not equal to the original category of an observation. The proportion of incorrectly classified entries is known as the False Classification Rate (FCR) and is calculated using the following equation.

(7) $$\begin{array}{r} \mathrm{FC}(X^{\left(\mathrm{imp}\right)},X^{\left(\mathrm{orig}\right)})=100\cdot \frac{1}{\sum_{i=1}^{n}\sum_{j\subseteq I_{\left(\mathrm{cat}\right)}}m_{\mathrm{ij}}}\sum_{i=1}^{n}\sum_{j\subseteq I_{\left(\mathrm{cat}\right)}}I(x_{\mathrm{ij}}^{\left(\mathrm{imp}\right)}\neq x_{\mathrm{ij}}^{\left(\mathrm{orig}\right)}) \end{array}$$

The notation $j\subseteq I_{\left(\mathrm{cat}\right)}$ indicates that only binary or categorical variables are considered.

### 3.3. Evaluation based on estimators

For these kinds of evaluations, we set the number of replications to 1000 in the simulations.

The bias of the estimate $\hat{\theta }$ is defined as the difference between the expected value of the estimate and true's population *θ*,

(8) $$\begin{array}{r} b(\hat{\theta })=E(\hat{\theta })-\theta . \end{array}$$

The estimator $\hat{\theta }$ is an unbiased estimator of *θ* if and only if $b(\hat{\theta })=0$.

The variance of an estimator is defined as

(9) $$\begin{array}{r} \mathrm{var}(\hat{\theta })=E\left[(\hat{\theta }-E[\hat{\theta }]{)}^{2}\right]. \end{array}$$

#### 3.3.1. (Root) mean squared error of parameter estimates

The MSE consists of the variance and the bias, $\mathrm{MSE}(\hat{\theta })=\mathrm{var}(\hat{\theta })+(b(\hat{\theta }){)}^{2}$. Often the root mean squared error is preferred, given by

(10) $$\begin{array}{r} \mathrm{RMSE}(\hat{\theta })=\sqrt{\mathrm{MSE}} \end{array}$$

The truth is only known in a simulation setup with synthetic data. Thus, the bias can only be computed in a simulation as well as the variance and (root) mean squared error.

#### 3.3.2. Coverage rate of confidence intervals

The coverage rate (CR) is the proportion of confidence intervals that capture the true value and should match the nominal rate. For instance, with a 95% confidence level, the simulated CR should be 0.95. A CR lower than the set confidence level suggests a biased estimate or insufficient spread of the imputed values. In contrast, a higher CR indicates an excessive spread of the imputed values, increasing the variability of the estimator.

### 3.4. Evaluation in a classification context

If one only intends to use the data for a classification task and only predictive performance is relevant, one can compare different imputation methods with the F1 score on a test set or via cross-validation. The F1 score is a widely used performance measure that remains informative, even in the case of unbalanced data. For a response with two categories, this is

(11) $$\begin{array}{rl} \mathrm{Accuracy} & =\frac{\mathrm{TP}+\mathrm{TN}}{\mathrm{TP}+\mathrm{TN}+\mathrm{FP}+\mathrm{FN}} \end{array}$$

(12) $$\begin{array}{rl} F1 & =\frac{2\mathrm{TP}}{2\mathrm{TP}+\mathrm{FP}+\mathrm{FN}} \end{array}$$

where

TP (true positive): An observation of the test data set that is correctly classified in relation to the reference group.

TN (true negative TN): An observation of the test data set that is correctly classified regarding the comparison group.

FP (false positive): An observation of the test data set that is incorrectly classified with respect to the reference group.

FN (false negative): An observation of the test data set that is incorrectly classified with respect to the comparison group.

## 4. Data sets and simulation setting

To evaluate methods, one can repeatedly remove some available observed values in simulated, fully observed real data sets, or observed parts of real data sets, predict those artificially created missing values with imputation methods and calculate the errors of the prediction, and average the obtained errors. Additional properties, such as the coverage rate of confidence intervals, can be investigated for simulated data. For all data sets where the results are based on one single realization, the seed of the random number generator was set to 123 for the Mersenne-Twister random number generator to remain as fair as possible.

### 4.1. Using real data

Where not defined differently, Robust Mahalonobis distances with a cut-off of $\chi_{\left(\mathrm{df}=p-1,p=0.99\right)}^{2}$ were used to determine outliers, with *p* the probability of being extreme based on the $\chi^{2}$ distribution with *df* the degrees of freedom that is the number of variables – 1. This threshold is used to avoid putting missing values in outlier observations. Missing values are never placed on outlier observations because otherwise a method would be judged to be working well if an outlier is imputed with an outlier value. In reality, the goal is rather to impute outliers with a reasonable value that is part of the distribution of the main part of the data set. Robustness is achieved by using the MCD estimator to calculate the covariance and the mean for the Mahalanobis distances ([see, e.g. 16]).

#### 4.1.1. Animals: average brain and body weights

For a simple illustration in 2D, we use the animals data set from [48]. The data consist of the average brain and body weights for 28 species of land animals. Data were logarithmic transformed prior to analysis. Ten missing values were inserted, and their position is clearly visible in Figure 1.

#### 4.1.2. Prestige: statistics on occupational groups

The Prestige data has 102 rows representing means in occupational groups and columns on education, income, percentage of women, occupational code, and type of occupation (three categories). This data set was analyzed (among others) in [18]. Note that results are comparable good for IRMI and missRanger using the original data. Differences in the methods show when adding two realistic occupational groups that moderately deviate from the occupational groups in the data set (*clerks* with education = 15, income = 1700, share of women = 0, prestige = 90 and type = professionals and *investors* with education = 10, income = 500,000, share of women = 0, prestige = 10 and type = professionals). Unless otherwise stated, 10% of the missing values in all variables were introduced with MCAR.

#### 4.1.3. Bushfire: satellite measurements

This data set contains satellite measurements on five frequency bands, corresponding to each of 38 pixels, and was used by [5] to locate bushfire scars. The data set is a complete data set consisting of 38 observations in 5 dimensions and was analyzed in [1,4,35,36,63]. The data set contains 12 clear outliers: 33–38, 32, 7–11; 12 and 13 are suspect [35,63]. The data set is available in R package robustbase [47].

#### 4.1.4. Freedman: crowding and crime in U.S. metropolitan areas

The Freedman data frame from the Bureau of the Census has 110 rows and 4 columns on crowding and crime in US metropolitan areas with 1968 populations of 250,000 or more. There are some missing data. The data was analyzed (among others) in [19] and is available in R package carData [20].

#### 4.1.5. Iris

The Iris data set is a multivariate dataset with 5 columns and 150 observations used and popularized in [17] and is one of the most commonly used data sets in statistics and statistics education. It contains the measurements sepal length and width and petal length and width for 50 flowers from each of 3 species of iris. The data set is available from the datasets package in R [44].

#### 4.1.6. Credit card fraud detection

The credit card fraud detection data set contains transactions, timestamps, a nominal label, and 984 observations on 30 features about the transactions [11]. The features are principal components of the original features due to confidentiality. The data set is available at Kaggle.

#### 4.1.7. Sonar

The sonar data set contains 208 observations on 61 sonar measurement features and a nominal class label, whether the object is a metal cylinder (mine) or a rock cylinder [23]. The data set is available at https://datahub.io.

### 4.2. Simulating data

Two different settings are used, one used in [45] and one that discusses misclassification. The aim is to show the influence of representative or non-representative outliers or extreme observations on imputations from different methods. All results produced in the different simulation settings are based on 1000 repetitions.

#### 4.2.1. Simulation setting 1: outliers

Raessler and Münnich [45] describes in detail how simulations can be used to determine whether a method of multiple imputation is correct or at least approximately correct, i.e. if the bias is zero and the coverage rate is equal to the nominal rate.

Let

(AGE,INCOME) ∼ N((401500)T,(104444300)2)

be the population from which samples of size 200 are drawn. Raessler and Münnich [45] set 30% of the income values to missing values. In the following, we only show results obtained with a MAR situation by increasing the probability of missing income information, the higher the AGE value.

We enhance the simulation study from [45] by including outliers and increasing the number of outliers from 0 to 80 to also observe the effects of the outliers on the methods. Outliers are simulated as high-leverage points by adding or subtracting the diagonal elements of the covariance matrix times 0.8 from the mean. In this way, the outliers were randomly placed at one of four positions with a variance of 1/5 of the original data.

As in our scenarios with real data, missing values are only inserted for observations that are not outliers.

#### 4.2.2. Simulation setting 2: misclassification

We simulate data as the relative energy consumption of a machine (per hour) depending on the runtime and the type of machine. With longer run times, it could become more efficient by heating up. Data generation is motivated by a real-world application with an industry partner.

(13) $$\begin{array}{r} \mathrm{energ}y_{i}=e^{2+I(\mathrm{typ}e_{i}=1)-0.1*\log(\mathrm{runtim}e_{i})+\epsilon_{i}} \end{array}$$

where

$$\begin{array}{r} \mathrm{typ}e_{i}∼\mathrm{Bin}(p=0.4),\;\mathrm{runtim}e_{i}∼\mathrm{EXP}(\lambda =1/100),\;\epsilon_{i}∼N(\mu =0,\sigma^{2}=0.1) \end{array}$$

with i=1,…,n=200.

As one would have to deal with in real life, some machines are misclassified to the wrong type in the resulting data set. As in the previous simulation setting, 0 to 80 machines are misclassified to show the effect on the imputation quality of the imputation methods. We add 30% missings in a MAR setting to the energy consumption for non-misclassified observations.

## 5. Results

### 5.1. Visual diagnostics in 2D

In Figure 1, the results for one realization of imputing the *Animals* data set are shown. Clearly, mean imputation gives the worst results, and all imputed values lie exactly on ${\bar{y}}_{\left(\mathrm{obs}\right)}$. One can observe that especially predictive mean matching with package mice, as well as midastouch and missRanger, is highly influenced by outliers. Also, mixgb and kNN might choose an outlier as a donor in a local neighborhood. The method IRMI is robust against outliers and provides by far the best results in terms of visual comparison of the tolerance ellipses but also on the precision measures MAPE, NRMSE, and MSECOR. For example, the mean absolute percentage error is only about half for IRMI compared to all other methods, and the covariance structure is perfectly preserved.

**Figure 2.**
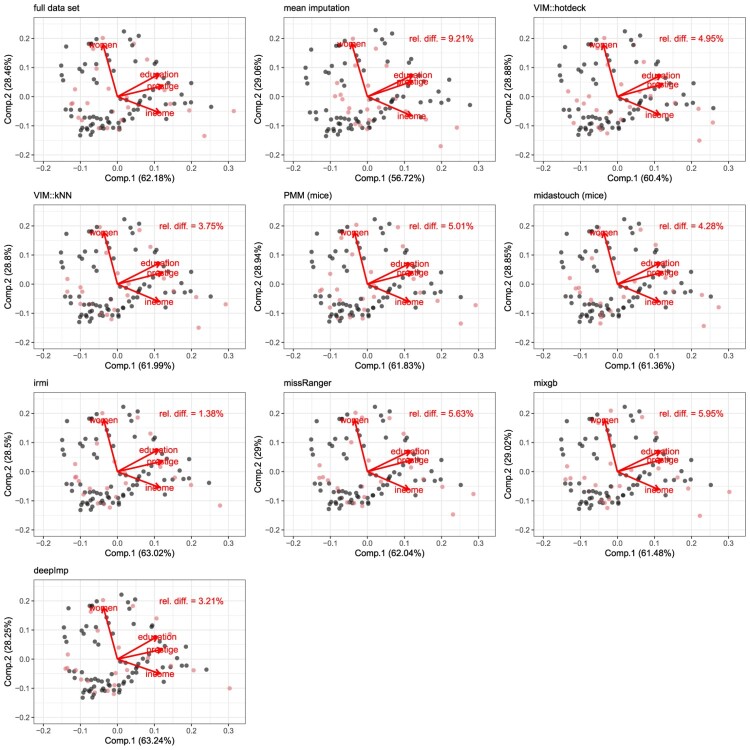
The biplot for the complete data set (Prestige) is shown in the upper left. The gray dots represent values that are set artificially to missing with MAR. The imputed values from different methods are also shown in gray, while the fully observed observations are again in black.

The scores and loadings of the first two principal components are shown in Figure 2. Above left is the result for the fully observed data set. From this data set, 25 missing values are inserted into the variable prestige with MAR related to income, as prestige is difficult to measure and therefore contains missing values. It is even slightly more difficult to determine prestige with high income, e.g. for general directors or ministers. The results represent the solution for one realization and can vary slightly for other realizations.

The relative difference (in percentage) of the angle of the loadings vectors is the smallest for IRMI, so the loadings are most similar to the *truth* for this method compared to any other method. Of course, IRMI robustly treats the outliers, so the outliers seen in the full original data below right are imputed differently. Of the model-based methods, PMM has the highest error, and the tree-based methods missRanger and mixgb have even slightly higher errors: obviously, the correlation between education and prestige is lower after imputation when applying these methods. Even hotdeck has approx. the same error as PMM, midastouch, missRanger and mixgb. deepImp and kNN outperform these methods.

### 5.2. Numerical results from real data

**Figure 3.**
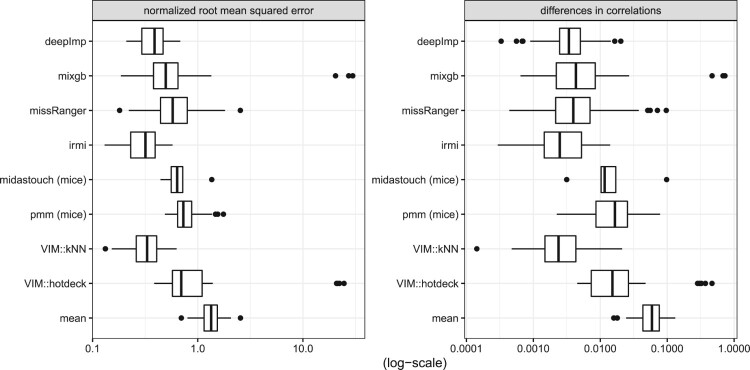
NRMSE and MSECOR from the imputation of the Prestige data.

Figure 3 shows the NRMSE and the differences in the correlation structure with respect to the data set *Prestige*. It can be seen that IRMI gives the best results, followed by kNN and deepImp. The results of missRanger and mixgb agree but are much worse than those of IRMI, kNN, and deepImp. midastouch, PMM, and hotdeck gave the worst results, except that mean imputation is the weakest imputation method.

**Figure 4.**
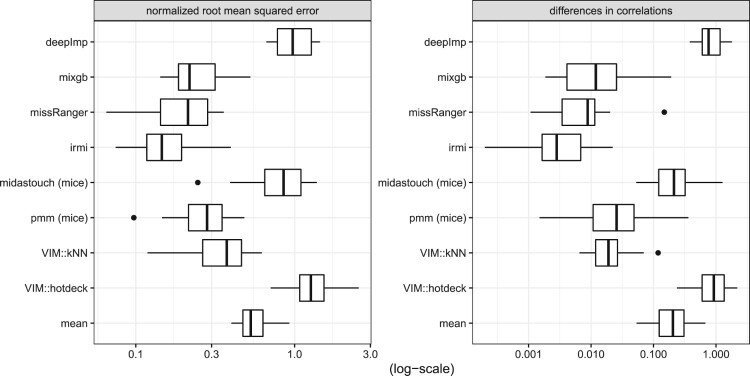
NRMSE and MSECOR from the imputation of the Bushfire data (in log-scale).

The imputation of the *Bushfire* data is done best by IRMI, see the results in Figure 4. This is not surprising since the *Bushfire* data includes a few outliers. missRanger performs better than mixgb. kNN and PMM gave comparable results. Midastouch, deepImp, and hotdeck imputation perform even worse than mean imputation. The reason why deepImp gave no promising results is both the small size of the data set and the outliers in the small data set.

**Figure 5.**
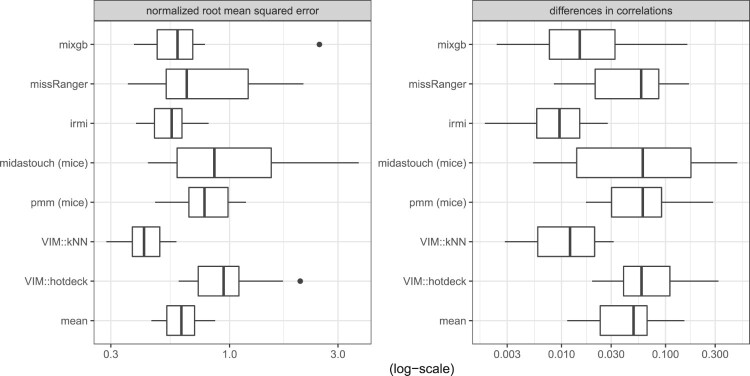
NRMSE and MSECOR from imputation of the Freedman data.

The best-performing methods for the *Freedman* data are kNN (for the NRMSE) and IRMI (for the MSECOR), see the results in Figure 5. mixgb now provides better results than missRanger. For missRanger and midastouch the results scatter a lot, while IRMI is more stable. Mean imputation is similar to missRanger, PMM, and midastouch. In other words, missRanger, PMM, and midastouch perform poorly on this data set.

**Figure 6.**
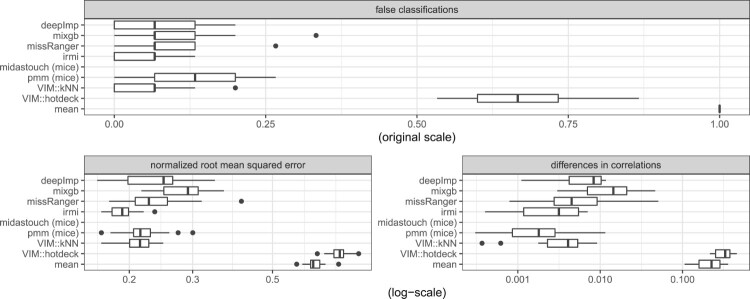
NRMSE, MSECOR (both in log10-scale) and false classifications of imputed values from the imputation of the Iris data.

Lastly, we show a result in a classification context using the well-known *Iris* data in Figure 6. For the numeric variables the NRMSE and MSECOR are calculated and displayed in log10-scale, and for the classification variable (*Species*) the ratio of false classifications of imputed values are reported. IRMI outperforms other methods, and, in addition, the results are mostly more stable than for other methods; e.g. the results on the NRMSE vary less for different runs than for other methods. The results for PMM and kNN agree. Also the results of the non-linear methods, missRanger, and deepImp agree, while mixgb is the worst non-linear method. Midastouch was unable to impute the missing values in the *Iris* data set. Therefore, no results can be reported.

### 5.3. Results from simulations

#### 5.3.1. Simulation setting 1: outliers

**Figure 7.**
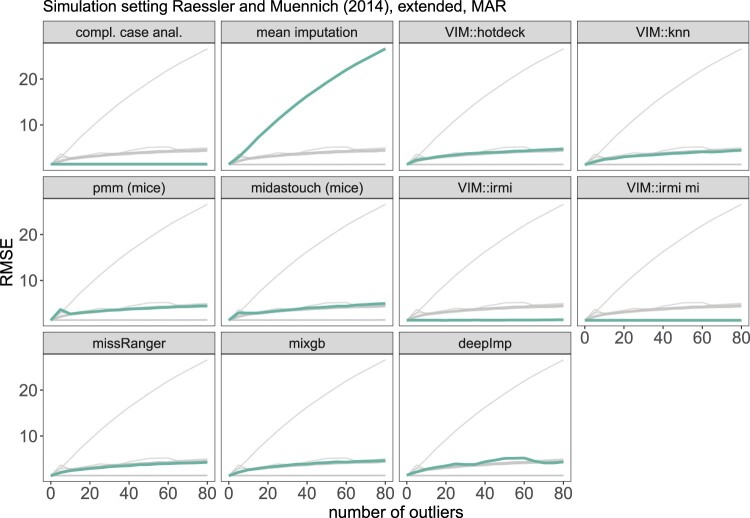
Results on the root mean squared error of the arithmetic mean estimates based on the simulation setting 1. The thick green line represents the actual method, while the thin light gray lines are used for comparison with other methods.

**Figure 8.**
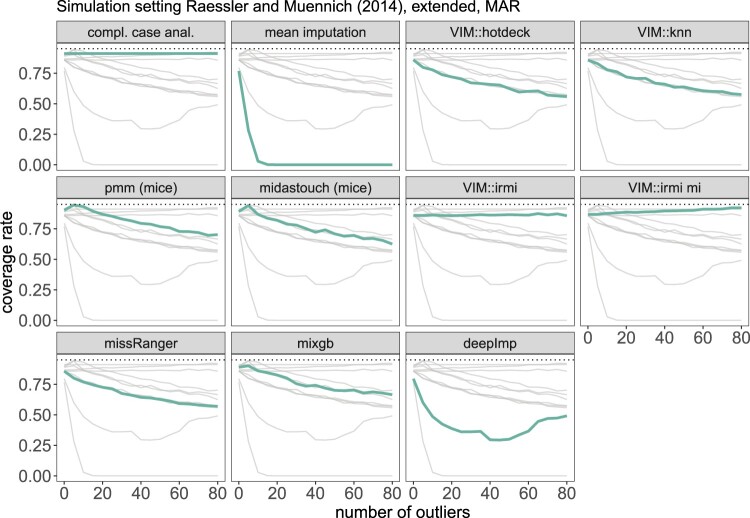
Results on the coverage rate of the confidence interval of the arithmetic mean based on the simulation setting 1. The thick green line represents the actual method, while the thin light gray lines are put for comparison with other methods.

The data are simulated as in Section 4.2.1, and the different imputation methods from Section 2 are applied. This is repeated 1000 times, and for each time, we calculate an estimated mean and 95% confidence interval for income. For every method and 0 to 80 outliers, we report the root mean squared error in Figure 7 and the coverage rate in Figure 8. Note that the outliers are only added for the imputation part. We are interested to see the effect of outliers on the imputation methods and not on the estimators of the population mean for income.

Almost all methods are influenced by outliers, so they can no longer perform meaningful imputations. Simple mean imputation performs the worst, and results are not even shown for more than ten outliers to make the other methods more comparable in the figure. VIM::knn and missRanger::missRanger outperform VIM::hotdeck, mice's PMM and midastouch, mixgb::mixgb and deepImp. Still, all these methods are very sensitive to outliers, and the imputations are highly distorted due to outliers in the data set. The corresponding RMSE in Figure 7 shows that the mean square error increases heavily, not to say *explodes*, already with few outliers in the data when imputation is performed with non-robust methods such as pmm (mice), missRanger, and all other non-robust methods. The imputations with IRMI are not affected by outliers: the RMSE remains at the same level regardless of how many outliers we add.

The coverage rates are comparable, with mice's pmm, and midastouch performing the best when no outliers are introduced. When outliers are introduced, the coverage rates of most methods change, clearly seen in Figure 8. The imputations of all non-robust methods are already affected by a few outliers, whereas robust imputation methods can handle even a large amount. A direct comparison of VIM::irmi (single imputation) and VIM::irmi mi (multiple imputation) shows a slightly better coverage rate when using IRMI with a multiple imputation framework.

Although the results from complete case analysis stay on a higher level, like for IRMI and IRMI mi, naturally deleting observations with missing values cannot affect imputations since no imputations are carried out. Thus in the case of outliers, a complete case analysis would be even better than all non-robust methods.

#### 5.3.2. Simulation setting 2: misclassification

**Figure 9.**
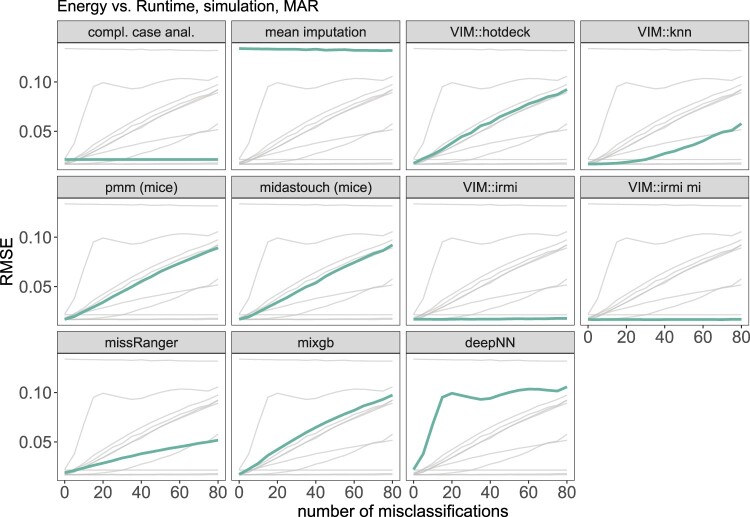
Results on the root mean squared error of the arithmetic mean estimates based on the simulation setting 2. The thick green line represents the actual method, while the thin light gray lines are used for comparison with other methods.

**Figure 10.**
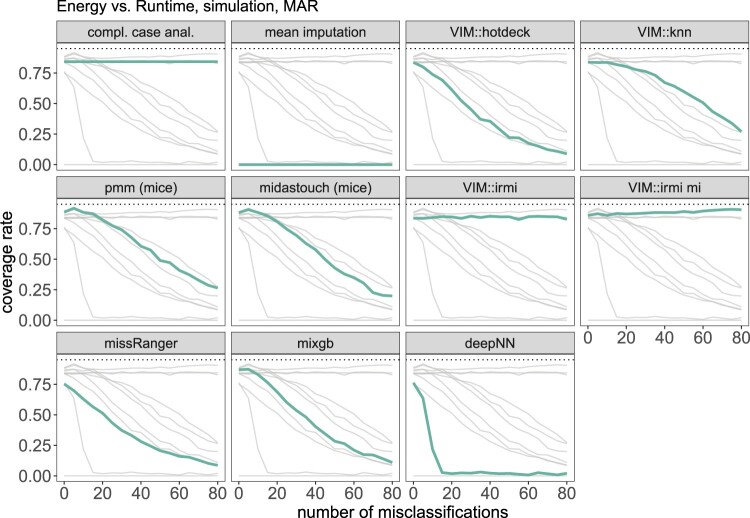
Results on the coverage rate of the confidence interval of the arithmetic mean based on the simulation setting 1. The thick green line represents the actual method, while the thin light gray lines are used for comparison with other methods.

Let us return to the example of the energy consumption of a machine explained in Section 4.2.2. Since the relationship between *energy* and *runtime* is log-normal, we first logarithmized these two variables, as is common practice, before imputation to apply an obvious transformation. The estimator of interest is the group mean of the *energy* of the first type of machine. As in the previous example, the data is simulated 1000 times for 0 to 80 misclassification, and different imputation methods are applied. For each imputation, we estimate the group mean of the *energy* of the first type of machine along with a 95% confidence interval. We report the root mean squared errors in Figure 9 and the coverage rates in Figure 10. As before, misclassifications are added only for imputations.

It is interesting to see that the second-best method is *k* nearest neighbor imputation (VIM::kNN), followed by missRanger, which gives reasonable imputations up to a relatively high number of misclassifications. Again, VIM::irmi, when applied in a single or multiple imputation setting, is robust against misclassification. deepNN provides the worst results (except mean imputation) and is incapable of having good coverage rates. Again, it is better to delete observations with missing values and perform a complete case analysis than to use non-robust imputation methods in case of misclassifications. However, the RMSE value is higher in that case than for robust imputation with IRMI, so IRMI is preferable.

Looking at Figure 10, we see the same patterns as in the previous example. mice's pmm, and midastouch result in the closest coverage rate to 95% in the case of very few misclassifications. When adding more, all the methods except for VIM:irmi and complete case analysis break down. When there are more than 10% misclassifications, one should better just delete missing values rather than applying non-robust imputation methods.

### 5.4. Results on classification

We might only be interested in the resulting classification performance if a dataset is only used to apply a classification algorithm. In the following two examples, we show real-world classification problems. At first, 10% of every feature is set to missing completely at random. The missings are then imputed using various methods and a random forest classifier is applied and tested on a hold-out test set. This procedure is repeated 100 times and the resulting F1 scores are reported.

#### 5.4.1. Credit card fraud

**Figure 11.**
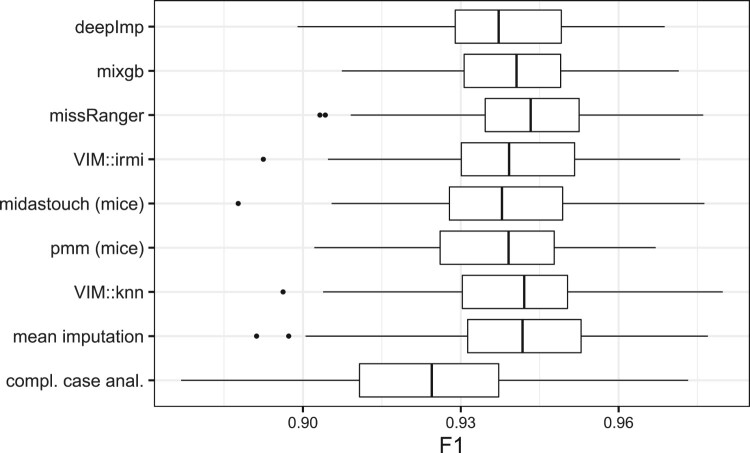
F1 score of a random forest classifier for the credit card fraud dataset after including and imputing missing with different imputation methods.

Using the credit card fraud dataset and the above-described process, the goal is to classify transactions as fraudulent or valid. Depending on the imputation method used, the performance of the random forest classifier changes and is shown in Figure 11. Basically, it does not matter which imputation method is used, except for complete case analysis that results in a lower F1 value. In other words: If one is only interested in classification performance, one can even use the otherwise poor mean imputation. Or if one is interested in comparing imputation methods, one should not use a classification example (only).

#### 5.4.2. Sonar

**Figure 12.**
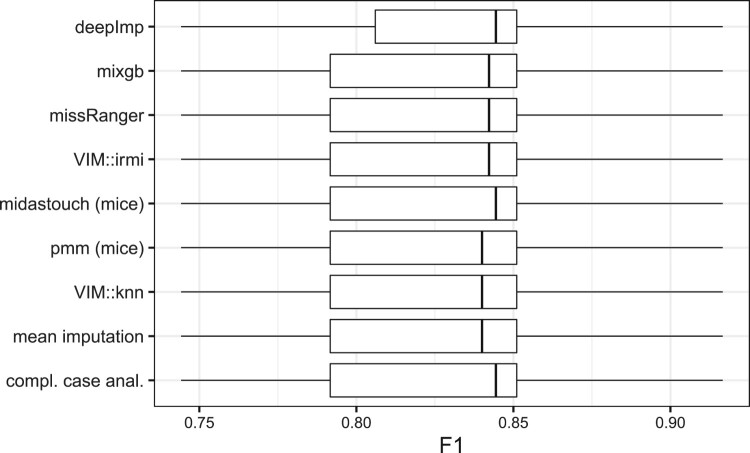
F1 score of a random forest classifier for the sonar dataset after including and imputing missing with different imputation methods.

Using the sonar data set and the above described process, a random forest classifier uses the sonar signals to classify rocks mines. The performance of the random forest depending on the imputation method is shown in Figure 12. We basically see the same patterns as in the credit card fraud example: it does not matter with which method the missing values are imputed.

### 5.5. Summary table of results and computational times

All results are summarized in rank order in the following tables. Table 1 shows that IRMI generally outperforms the other methods in all assessment metrics considered and in visualization diagnostic plots. kNN is mostly second place and performs well compared to the other methods. missRanger also performs well, with weak results, except for biplot analysis and coverage. deepImp scores well on visualization, precision and correlation measures, but performs less well on measures based on the variance of the estimators. mixgb is mostly outperformed by missRanger. PMM and midastouch are ranked below average. Mean imputation performs worst in all evaluation measures.

**Table 1.** Summary of the results: ranking of methods according to the quality of imputations for all considered different kinds of evaluation criteria.

Rank	Vis 2D	Biplot	Precision	Correlation	RMSE	Coverage	F1
1	IRMI	IRMI	IRMI	IRMI	IRMI	IRMI	*
2	midastouch	deepImp	missRanger	kNN	kNN	kNN	*
3	missRanger	kNN	deepImp	missRanger	missRanger	PMM	*
4	deepImp	midastouch	kNN	PMM	midastouch	midastouch	*
5	kNN	hotdeck	mixgb	deepImp	PMM	mixgb	*
6	PMM	PMM	PMM	mixgb	hotdeck	hotdeck	*
7	mixgb	missRanger	midastouch	hotdeck	mixgb	missRanger	*
8	hotdeck	mixgb	hotdeck	midastouch	deepImp	deepImp	*
9	mean	mean	mean	mean	mean	mean	*

Notes: * …the results are too close to each other, a ranking is therefore not meaningful.

Table 2 reports the rankings for each data set. IRMI outperforms all other methods, except for the Freedman data where kNN was better. Again kNN mostly is on second place. PMM and midastouch gives unsatisfactory results for some of the data sets (Prestige, Bushfire, Freedman, Credit Fault), but they perform well – compared to other methods - for the Income data. deepImp is sensitive to outliers and misclassifications, thus the results are worst for Bushfire and Energy data. missRanger is sometimes ranked very badly, e.g. for Income and Energy data.

**Table 2.** Summary of the results: ranking of methods according to the quality of imputations for all considered data sets.

Method	Animals	Prestige	Bushfire	Freedman	Iris	Income	Energy	Credit Fraud	Sonar
mean	9	9	6	4	8	9	9	1	2*
hotdeck	8	8	9	8	7	6	5	^-^	^-^
kNN	5	2	5	1	2	2	2	3	2*
PMM	6	7	4	6	3	2	3	6	2*
midastouch	2	6	7	7		2	3	6	2*
IRMI	1	1	1	2	1	1	1	3	2*
missRanger	3	5	2	5	5	7	7	1	2*
mixgb	7	4	3	3	6	5	5	5	2*
deepImp	4	3	8		4	8	8	6	1

Notes: ^-^ …was not tested due to the absence of an ordering variable in the data set. * …the results are too close to each other, therefore, a ranking is not questionable.

The computational times are reported in Table 3. Note that only default settings are used, and the computational times can vary depending on how these defaults are overwritten. mixgb and deepImp are the slowest, followed by PMM and midastouch.

**Table 3.** Computational times in seconds on Intel Core i9 with 64 GiB memory, single CPU.

Method	Animals	Prestige	Bushfire	Freedman	Iris	Credit Fraud	Sonar
mean	0.1	0.1	0.1	0.1	0.5	0.1	0.1
hotdeck	4.2	4.4	4.2	4.2	15.0	4.1	4.1
kNN	3.4	3.4	3.3	3.2	20.6	3.4	3.4
PMM	17.8	17.2	17.4	17.8	169.8	17.1	17.6
midastouch	28.5	27.8	28.0	27.6	395.8	26.5	26.7
IRMI	10.2	10.2	10.0	10.1	84.9	9.8	9.9
missRanger	16.6	16.9	16.7	16.4	118.4	16.3	17.7
mixgb	227	248	234	160	1786	180	258
deepImp	2208	2306	2322	2241	49,557	2231	2492

## 6. Discussion

Jadhav *et al.* [28] compared the methods of mean imputation, median imputation, kNN imputation, predictive mean matching (PMM), linear Bayesian regression, linear regression, non-Bayesian method and random sampling with numerical data sets from the UCI machine learning repository. They used the normalized RMSE (NRMSE) to compare the performance of the different imputation methods. kNN imputation [32] outperformed the other methods regardless of the number of missings inserted. This is comparable to our results, as kNN imputation often leads to better results compared to the examined methods of [28]. However, we examined more methods, used many more evaluation metrics and methods, and data sets that included non-numeric variables. IRMI was not studied by [28], but it was the superior method in our comparisons.

Also others compared numeric data with only very few selected methods only, like [54]. They found out that missForest should not be used for imputation since it gave the worst results compared to imputation with principal component analysis and similar methods. However, their simulations were based on data generated with the multivariate exponential power and the multivariate skew-normal distribution only, thus under ideal considerations and for numeric data only. We did not consider imputation methods based on principal component analysis because they (imputePCA and MIPCA from R package missMDA, impPCA from package VIM) did not give competitive results for our numerical data under investigation and are only suitable for numerical data.

In other studies, one of the problems is data generation in simulation studies, such as [25] who used a multivariate normal distribution of the covariate data. This is of interest for comparing methods in an idealized setting, but does not reflect whether the imputation methods produce reliable results for real or realistic data, as was investigated in our paper.

Furthermore, the simulation study of [42] is based on simplified data generation based on normal distributions and the choice of only one single evaluation metrics: the mean squared error between the original data values and the imputed ones. Their simulation results indicate that (group) mean imputation performed better compared to MICE which had the lowest performance. This contradicts our results and can only be explained by the fact that the simulation is based on oversimplified, non-realistic assumptions and that the evaluation metric does not take into account the variances of the estimators and coverage rates, but only represents a precision measure.

May *et al.* [38] compared with mice (using PMM for continuous variables and logistic regression for categorical variables), missForest and kNN (from VIM) for mixed-type data and found that imputation with random forests was superior, while PMM (mice) performed worst. The results were obtained by deleting and imputing the values of all the cases, and the performance was assessed by comparing the original and imputed values with the mean square error. Again, this covers only one aspect, and this precision measure does not assess the variance of the estimators or other non-precision measures as we additionally applied in this contribution.

Woznica and Biecek [68] compared random sample, mean imputation, softImpute (10) that works only for numeric variables, missForest, kNN (from package VIM), hotdeck (also from package VIM in two versions, sequential, random hot-deck), PMM and logistic regression (with package mice) in a classification context. According to the averaged ranking, mean imputation was best for 2 of 5 models. Mean imputation was the winner for the F1 measure and kNN for the AUC measure. This is consistent with our results, as in the classification context, the choice of imputation method did not matter much, and even mean imputation is competitive in this context. However, we did not only investigate classification performance, and in all other experiments and simulations we showed that mean imputation performs (by far) the worst.

Jäger *et al.* [29] also found that simpler imputation methods in a classification context based on the RMSE and F1 measure gave competitive results. They also compared mean and kNN imputation with missForest, variational autoencoders, and generative adversarial network imputation. In almost all experiments, random forest-based imputation achieved the best imputation quality, but as mentioned earlier, the differences with mean imputation were very small. VAE and gain failed frequently and were therefore not competitive. This is also in line with our results on classification, but since we compared with methods such as IRMI, deepImp, mixgb and many others and did not only compare the methods in a classification context based on RMSE and F1 measures, we can greatly extend their conclusions and point out that other methods – such as IRMI - gave significantly better results.

Overall, we compare more methods under more realistic assumptions with a wide range of different evaluation measures – from visual diagnosis to precision measures to coverage rates and misclassification errors for real and simulated data. In this way, a detailed judgement about the performance of imputation methods can be made about the advantages and disadvantages, and recommendations can be formulated, as is done in the next section.

## 7. Conclusion

Our research expands existing literature by comparing common imputation methods under realistic scenarios involving outliers, misclassifications, and model errors, using various evaluation measures. While visualization tools like [59] help in spotting poor imputations, they have limitations, especially in MAR situations. Biplots, however, reveal multivariate data structures, aiding in visual comparison of imputation methods. Precision measures assess the accuracy of imputation methods but don't fully address imputation and model uncertainty, which are better evaluated by RMSE or coverage rates. Interestingly, mean imputation often performs well in misclassification scenarios, but relying solely on classification performance is inadequate as data have broader uses. Evaluating imputation methods on artificial data can assess coverage rates effectively, yet may not always reflect real data scenarios, hence the importance of testing on actual datasets by artificially marking some values as missing. Care should be taken to avoid imputing outliers with outlier values. While all investigated methods handle mixed variable types, not all are suitable for special distributions like semi-continuous or zero-inflated data, as summarized in Table 4.

**Table 4.** Rough comparison between methods for specific situations in a data set.

Method	Mixed type of variables	Individual user-specified models	Outliers	Misclassifications	MAR	Multiple missingness	Non-linearities	Multiple imputation
mean/median/ mode	✓		(✓)	(✓)		✓		
hotdeck (VIM)	✓				✓	✓	✓	
kNN (VIM)	✓				✓	✓	✓	
PMM/log-reg/polyt. (mice)	✓				✓	✓		✓
midastouch/log-reg/polyt. (mice)	✓				✓	✓		✓
IRMI (VIM)	✓^+^	✓	✓	✓	✓	✓		✓*
missRanger	✓	**			✓	✓	✓	✓
mixgb	✓	**			✓	✓	✓	✓
deepImp	✓	**			✓	✓	✓	(✓)***

Notes: * only type 1. ** not applicable since it is a non-linear model. *** only approx./limited through dropout. ^+^ can deal also with semi-continuous variables.

IRMI offers several advantages, including handling semi-continuous variables and allowing for separate models for each variable, unlike other multivariate imputation methods that use a response-versus-predictors approach. Particularly beneficial for real data, which often contain outliers or misclassifications, robust imputation provides an effective tool without requiring expertize in outlier detection. These methods cleverly reduce the influence of outliers and misclassifications, ensuring limited impact on the imputation process. Thus, robust imputation methods yield comparable results to non-robust ones in elliptically shaped data and remain unaffected by outliers and misclassifications, making them a reliable choice for practical data imputation.

Relationships between variables are often non-linear, but can usually be linearized through transformations (like logarithmic scaling) or feature additions (such as quadratic terms or indicators for structural breaks). In practice, it's advisable to first establish linear relationships before imputing raw datasets, except with non-linear methods like deepImp, random forests, or boosting techniques. These methods, including missRanger and mixgb, are effective in avoiding model misspecifications, particularly in datasets without outliers and where no additional variable transformation is desired. However, automated imputation methods like the multivariate framework of mice may not always check model assumptions, making outlier and misclassification detection time-consuming. Using robust imputation procedures like IRMI, which downweight the influence of outliers, is a preferred alternative to prevent imputations from being arbitrarily skewed by such anomalies. kNN imputation provides reliable results that are often almost as good as IRMI, but often better than missRanger, mixgb, deepImp, PMM, midastouch. It is an attractive method, even if multiple imputation is not (easily) possible. It is easy to explain and does not involve a statistical model, so no misspecifications of a statistical model can occur.

The multiple imputation paradigm guarantees that the variances of the estimators are not underestimated and can reflect the uncertainty of the model and the imputation. Model uncertainty can be considered by bootstrapping the data set or by Bayesian regression, while imputation uncertainty is achieved by drawing from predictive distributions. While the mice framework considers both model and imputation uncertainty, IRMI only considers imputation uncertainty by default. Model uncertainty can be considered by bootstrapping the data before each (repeated) call of IRMI or by using its experimental extension in [57]. Nevertheless, in real-life situations, model mispecifications, outliers, and misclassifications have more influence on the imputations, as easily can be seen in the results presented in this paper.

VIM::irmi used as a single imputation method, but also VIM::irmi used in a multiple imputation framework are robust to outliers and misclassifications and provide reasonable imputations even in the presence of outliers and misclassifications.

Even a few misclassifications can severely affect non-robust imputation methods. In this case, even a complete case analysis would be more reliable than using non-robust methods. Robust imputation can handle misclassifications and is the best choice.

## Data Availability

All data sets underpinning the findings of this study are freely available on the Comprehensive R Archive Network (CRAN) and Kaggle. Details of the corresponding data repositories can be found in Section 4 together with the data description.
